# Levels of Pathogenic Th17 and Th22 Cells in Patients with Rheumatoid Arthritis

**DOI:** 10.1155/2022/5398743

**Published:** 2022-08-13

**Authors:** Marlen Vitales-Noyola, Berenice Hernández-Castro, Diana Alvarado-Hernández, Lourdes Baranda, Sofía Bernal-Silva, Carlos Abud-Mendoza, Perla Niño-Moreno, Roberto González-Amaro

**Affiliations:** ^1^Sections of Molecular and Translational Medicine and Medical Genomics, Research Center of Health Sciences and Biomedicine (CICSaB), UASLP, San Luis Potosí, SLP, Mexico; ^2^Departments of Immunology and Microbiology, School of Medicine, UASLP, San Luis Potosí, SLP, Mexico; ^3^Regional Unit of Rheumatology and Osteoporosis, Hospital Central Dr. Ignacio Morones Prieto, San Luis Potosí, SLP, Mexico; ^4^Laboratory of Genetics, School of Chemical Sciences, UASLP, San Luis Potosí, SLP, Mexico

## Abstract

Rheumatoid arthritis (RA) is a chronic autoimmune condition characterized, among others, by tissue damage and activation/differentiation of proinflammatory lymphocytes. Accordingly, several studies have concluded that type 17 T helper (Th17) cells seem to have an important role in the pathogenesis of this condition. However, the strategy for the identification and analysis of proinflammatory Th17 cells in those studies has not been consistent and has usually been different from what was originally described. Therefore, we decided to evaluate the levels of Th17 cells in patients with RA employing an extended immune phenotype by using an eight-color multiparametric flow cytometry analysis. For this purpose, blood samples were obtained from 30 patients with RA and 16 healthy subjects, and the levels of Th17 and type 22 helper (Th22) lymphocytes were analyzed as well as the *in vitro* differentiation of peripheral blood mononuclear cells into Th17 lymphocytes induced by interleukin-23 (IL-23) and IL-1*β*. We found significant enhanced levels of total Th17 lymphocytes (defined as CD4^+^IL-17^+^) as well as enhanced numbers of their pathogenic (defined as CD4^+^CXCR3^+^IL-17^+^IL-22^+^CD243^+^CD161^+^IFN-*γ*^+^IL-10^−^) and nonpathogenic (CD4^+^CXCR3^+^IL-17^+^IL-22^−^CD243^−^CD161^−^IFN-*γ*^−^IL-10^+^) cell subsets in patients with RA. Likewise, the number of Th22 (CD4^+^CXCR3^+/-^IL-17^−^IL-22^+^) was also increased in these patients compared to healthy controls. However, the *in vitro* induction/differentiation of pathogenic Th17 cells showed similar results in controls and patients with RA. Likewise, no significant associations were detected in patients with RA between the levels of Th17 or Th22 cells and clinical or laboratory parameters. Our data indicate that patients with RA show enhanced blood levels of the different subsets of Th17 cells and Th22 lymphocytes tested in this study and suggest that these levels are not apparently associated with clinical or laboratory parameters.

## 1. Introduction

Rheumatoid arthritis (RA) is a chronic autoimmune systemic inflammatory disease that mainly affects the diarthrodial joints. T lymphocytes have an important role in the pathogenesis of this condition, which is characterized by chronic synovial inflammation, cartilage destruction, and juxta-articular bone loss, leading eventually to joint destruction [1]. Thus, it has been widely described that different T cell subsets, mainly type 1 and 17 helper (Th1 and Th17, respectively) lymphocytes as well as cytokines and chemokines, are involved in the pathogenesis of the tissue damage seen in RA [1, 2]. Accordingly, it has been reported that the levels in the peripheral blood of different T cell subsets are associated with the activity of the disease or the therapeutic response to disease modifying antirheumatic drugs [3–6]. However, the strategy for the identification and analysis of proinflammatory Th17 cells in these studies has not been consistent, and discordant data have been reported [3–8].

Type 17 T helper (Th17) cells were described since 2005 and are characterized by the synthesis of IL-17 and the expression of the ROR*γ*t transcription factor [9]. These lymphocytes have an important role in the immunity against different pathogens, including extracellular bacteria and fungi [9, 10]. In addition, it has been widely described in the involvement of this lymphocyte subset in the pathogenesis of several immune-mediated inflammatory diseases such as multiple sclerosis, psoriasis, autoimmune thyroid disease, inflammatory bowel disease, and rheumatoid arthritis (RA) [9, 11–14]. However, it has also been described that Th17 cells seem to exert a relevant homeostatic role in different tissues and the immune system [15]. Accordingly, the blockade of IL-17 or its receptor with therapeutic monoclonal antibodies (MoAb) has provided some unexpected results, with no significant effect in Crohn's disease but encouraging results in multiple sclerosis [16, 17]. However, it has been reported, in an animal model of RA, that the administration of an anti-CXCR3 MoAb (a chemokine receptor that is expressed by different immune cells, including Th17 lymphocytes) significantly reduces the inflammation and articular damage, with diminution of proinflammatory mediators (e.g., TNF-*α*, interferon-*γ* (IFN-*γ*), IL-17A, and IL-22) and increased levels of Foxp3+ T regulatory (Treg) cells [2]. These apparently contradictory data seem to be explained by the existence of different Th17 cell subsets as well as by their plasticity [18–21]. Thus, pathogenic and nonpathogenic Th17 (pTh17 and npTh17, respectively) lymphocytes have been described as well as their transdifferentiation into Th1-like and Treg cells [18, 20, 22].

Pathogenic Th17 cells (also called as Th17.1, ex-Th17, and nonclassical Th1 cells) are characterized by the synthesis of IL-17A, IFN-*γ*, and GM-CSF as well as by the expression of the T-bet transcription factor and the chemokine receptors CXCR3 and CCR6 [23, 24]. These lymphocytes have an important role in the pathogenesis of RA, exerting a proinflammatory and osteoclastogenic effect in the synovial membrane and the juxta-articular bone [9, 13, 25]. In this regard, increased levels of pTh17 lymphocytes have been detected in the synovial membrane and peripheral blood of patients with RA, and it has been reported that these levels are associated with disease activity and other clinical features of the disease [3, 4, 6, 7]. Likewise, it has been proposed that the levels of pTh17 cells in the peripheral blood from these patients could be a useful marker to predict the therapeutic response to the T cell costimulation blocker abatacept [4] or to follow-up the treatment with other biological agents [5]. However, although the full phenotype of pTh17 cells was defined several years ago [23], in the association studies between the levels of these cells and clinical features in patients with RA, different flow cytometry strategies (and, therefore, distinct cell phenotypes) for their identification have been employed [3–9]. In addition, it has been reported that different Th17 cell subsets, including pTh17 and np-Th17 cells, seem to have a similar capability to induce the *in vitro* activation of synovial fibroblasts [25, 26].

Type 22 T helper (Th22) cells are characterized by synthesis of IL-22 (and also IL-13 and TNF-*α*) but not IL-17 or IFN-*γ* [27]. These lymphocytes, in addition to their role in host defense against different infectious agents and homeostasis, may also exert a proinflammatory effect, participating in the pathogenesis of different immune-mediated conditions such as psoriasis, asthma, inflammatory bowel disease, and systemic sclerosis as well as in viral infections [27, 28]. In addition, Th22 and IL-22 seem to have a relevant role in the pathogenesis of RA [29, 30]. Accordingly, it has been reported that the combination of methotrexate and leflunomide is able to target Th22 cells, accounting, at least in part, for their disease-modifying therapeutic effect in RA [30].

The aim of this study was to further evaluate the blood levels of Th17 cell subsets and Th22 lymphocytes in patients with RA by using an eight-color multiparametric flow cytometry analysis. We detected increased levels of most of these immune cells in the peripheral blood from RA patients, with no apparent association with laboratory or clinical features of the disease. These data suggest that these immune cells and their subsets have a complex role in the pathogenesis of RA and other chronic inflammatory conditions.

## 2. Materials and Methods

### 2.1. Patients and Healthy Controls

Peripheral blood samples were obtained from 30 patients with RA and 16 healthy individuals, the main characteristics of which are shown in Table 1. Mean age and the female-to-male ratio were similar in both groups. Patients were classified, according to the Disease Activity Score 28-joint counts (DAS28) as those with high (DAS28 > 4.0) and low (DAS28 ≤ 4.0) activity. Likewise, patients were also classified as seropositive and seronegative, according to their serum levels of rheumatoid factor (RF) and anticyclic citrullinated peptide (ACCP) antibodies. All patients were under therapy with disease-modifying antirheumatic drugs, eleven with low-dose methotrexate alone (MTX, 7.5-10.0 mg/week), eleven with MTX (7.5-12.5 mg/week) plus prednisone (PDN, 2.5-7.5 mg/day), and eight with MTX (7.5-10.0 mg/week) plus PDN (2.5-5.0 mg/day) and sulfasalazine (SSZ, 1.5-2.0 g/day). In all these patients, no evidence of other chronic or acute diseases was found at the time of the study, and none of them were under therapy for any condition other than RA. Moreover, in this study, healthy controls corresponded to clinically healthy individuals, with no evidence of any chronic or acute disease, who were not under therapy for any condition. All patients and controls signed a written informed consent, and this study was performed according to the Declaration of Helsinki. This study was approved by the institutional Bioethical Committee.

### 2.2. Flow Cytometry Analysis of Th17 and Th22 Lymphocyte Subsets

Peripheral blood mononuclear cells (PBMC) were isolated by Ficoll–Paque PLUS (GE Healthcare, Uppsala, Sweden) density-gradient centrifugation. Cell viability was assessed by trypan blue dye exclusion, and it was always higher than 95%. Cells were resuspended in BD Horizon Brilliant Stain Buffer (BD Life Sciences, San Jose, CA) and stained with the following MoAbs: anti-CD4-BV510 (BD Horizon, San Jose, CA), anti-CD183 (CXCR3)-APC-Cy7 (BioLegend Inc., San Diego CA), anti-CD243 (MDR-1)-PerCp/Cy5.5 (BioLegend), and anti-CD161-PE/Cy7 (BioLegend). Then, cells were fixed, permeabilized with p-formaldehyde 4.0% and saponine 0.1%, and additionally stained with the following MoAbs: anti-IL-10-PE (BioLegend), anti-IFN-*γ*-FITC (BioLegend), anti-IL-17A-BV421 (BD Horizon), and anti-IL-22-APC (eBioscience Inc., San Diego, CA). Negative controls were designed according to the fluorescence minus-one (FMO) strategy, and cells were analyzed in a FACSCanto flow cytometer (Becton–Dickinson) and with the Flow Jo software (Tree Star Inc, Ashland, OR).

### 2.3. In Vitro Induction of Pathogenic Th17 Cells

PBMC from RA patients were stimulated with MoAbs against CD3/CD28 (5.0 ug/ml in both cases, plate-bound) in IMDM culture medium (Gibco, Grand Island, NY), supplemented with 10% fetal bovine serum, glutamine (2.0 mM), and penicillin (100 u/ml)/streptomycin (100 *μ*g/ml), and incubated at 5% CO_2_ and 37°C for 6 days. In order to induce the differentiation of pathogenic Th17 cells, human recombinant IL-23 (R&D Systems, Minneapolis, MN, 10.0 ng/ml, or this cytokine plus IL-1*β* (Peprotech, Rocky Hill, NJ, 8.0 ng/ml) was added, at days 1 and 3 of cell culture. Three hours before harvesting, cells were incubated with the leukocyte activation cocktail (PMA plus Ionomycin) and GolgiPlug (BD Pharmingen), and then cells were stained and analyzed by flow cytometry, as described above.

### 2.4. Statistical Analysis

Data with normal distribution were represented as the arithmetic mean and SD, and data with a non-Gaussian distribution were represented as the median and interquartile range (*Q*_1_-*Q*_3_). Comparisons of two groups were analyzed with the Student *t* or the Mann–Whitney *U* tests. Comparisons among three groups were analyzed by the Kruskal-Wallis nonparametric test, with Dunn's posttest. The association between two variables was determined with the Spearman or Pearson correlation tests. Data were analyzed using the GraphPad Prism software version v8.0.1 (San Diego, CA), and *p* values <0.05 were considered as significant.

## 3. Results

### 3.1. Increased Levels of Th17 and Th22 Cells in RA Patients

**Figure 1:**
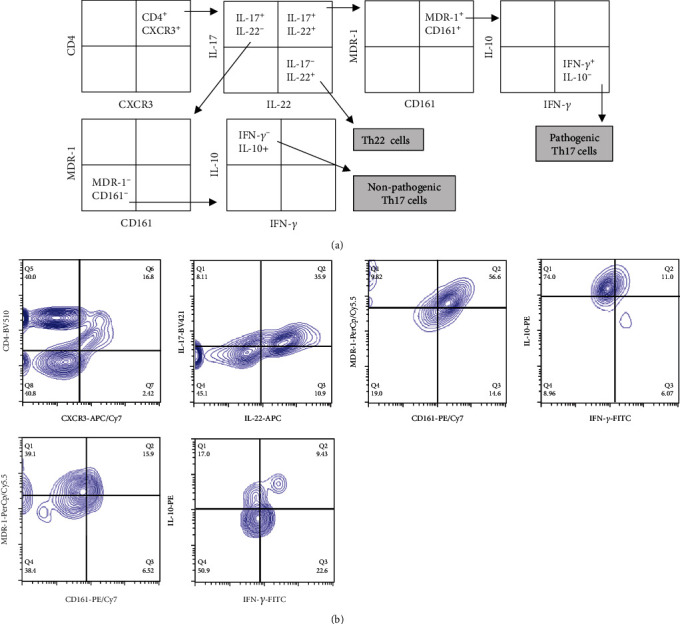
Flow cytometry strategy for the analysis of Th17 and Th22 cells. (a) PBMC were labeled with MoAbs against CD4, CXCR3, IL-17, IL-22, CD243 (MDR-1), CD161, IFN-*γ*, and IL-10 and analyzed by multiparametric flow cytometry, as indicated. This analysis allowed to detect the cell subsets of interest, pathogenic Th17 cells, nonpathogenic Th17 cells, and Th22 lymphocytes. (b) Flow cytometry images of a representative analysis of a blood sample from a healthy donor are shown. The type of fluorochrome coupled to each antibody is indicated, and the negative stain controls were selected according to the fluorescence minus one (FMO) strategy.

**Figure 2:**
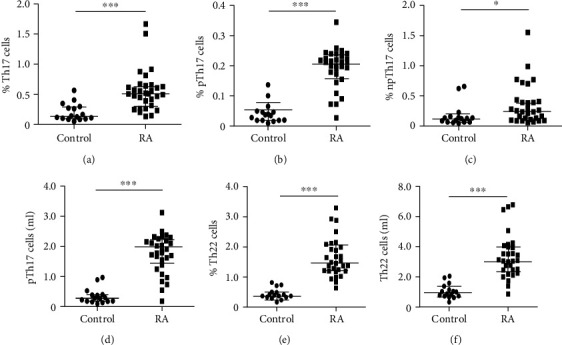
Levels of Th17 and Th22 lymphocytes in patients with RA. Blood samples from 30 patients with rheumatoid arthritis (RA) and 16 healthy controls (Control) were obtained and PBMC were isolated, stained, and analyzed by multiparametric flow cytometry, as stated in Materials and Methods and shown in Figure 1. (a) Percentages of CD4^+^IL-17^+^ cells, referred to total lymphocytes. (b) Percentages of pathogenic Th17 (pTh17) cells referred to total lymphocytes. (c) Absolute numbers of pTh17 cells/ml. (d) Percentage of nonpathogenic Th17 (npTh17) cells, referred to total lymphocytes. (e) Percent of Th22 cells (CD4^+^CXCR3^+/-^IL-17^−^IL-22^+^), referred to total lymphocytes. (f) Absolute numbers of Th22 lymphocytes/ml. Horizontal lines correspond to the median and *Q*_1_-*Q*_3_ interquartile range. ^∗^*p* < 0.05, ^∗∗∗^*p* < 0.005 (Mann–Whitney *U* test).

The absolute number and percentage of Th17 lymphocytes in blood samples from patients with RA and healthy controls were analyzed by multiparametric flow cytometry, according to the strategy shown in Figure 1. According to this, we detected a significant increased proportion of total Th17 (CD4^+^IL-17^+^) cells in patients with RA compared to healthy controls (*p* < 0.005, Figure 2(a)). Likewise, RA patients showed significant higher levels of both, pathogenic (CD4^+^CD183^+^IL-17^+^IL-22^+^CD243^+^CD161^+^IFN-*γ*^+^IL-10^−^) (*p* < 0.005, Figure 2(b)) and nonpathogenic (CD4^+^CD183^+^IL-17^+^IL-22^−^CD243^−^CD161^−^IFN-*γ*^−^IL-10^+^) (*p* < 0.05, Figure 2(c)) Th17 lymphocytes, compared to healthy controls. Similar results were observed when data were analyzed as the absolute number of cells, with significant differences in the case of total Th17 cells (CD4^+^IL-17^+^, *p* < 0.05, data not shown), pTh17 lymphocytes (*p* < 0.005, Figure 2(d)), and its nonpathogenic counterpart (*p* < 0.05, data not shown). Moreover, the levels of Th22 lymphocytes (CD4^+^CXCR3^+^IL-17^−^IL-22^+^) were also significantly increased in patients compared to controls, both, their percentages (*p* < 0.005, Figure 2(e)) and their absolute numbers (*p* < 0.005, Figure 2(f)). Similar results were observed when CD4^+^CXCR3^−^IL-17^−^IL-22^+^ lymphocytes were analyzed, either as their proportion or absolute number (*p* < 0.05 in both cases, RA compared to healthy controls, data not shown). A summary of the comparisons and *p* values of the different Th cell subsets analyzed in this study is shown in Table 2.

### 3.2. Levels of Th17 and Th22 Cells Are Not Apparently Associated with Clinical or Laboratory Parameters

**Figure 3:**
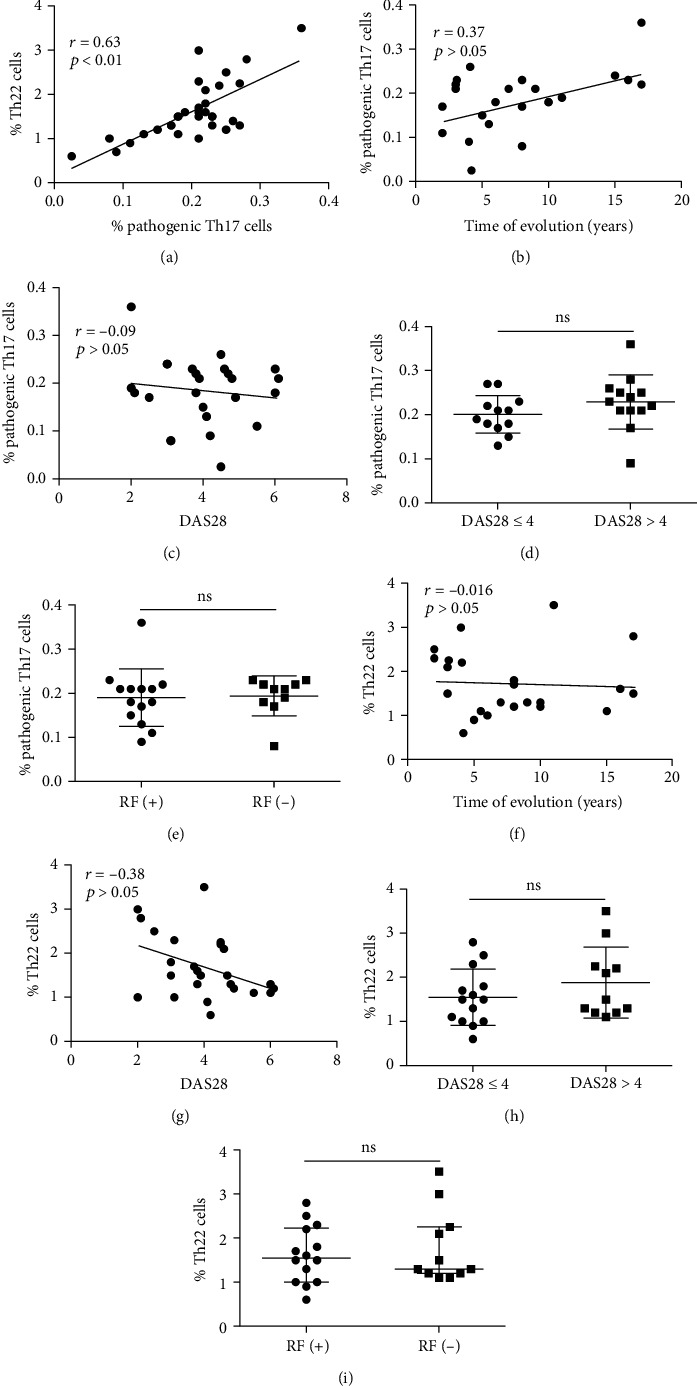
Association analysis between the levels of Th17 lymphocytes or Th22 cells and clinical parameters in RA patients. The correlation analysis between the percentages of pathogenic Th17 (pTh17) and Th22 cells (a), the percentage of pTh17 cells and time of evolution of disease (b), the percentage of pTh17 cells and the Disease Activity Score 28 (DAS28) (c), the percentage of Th22 cells and time of evolution of disease (f), or DAS28 (g) is shown. *r* and *p* values (Spearman sum rank test) are shown. The comparisons between the levels of pTh17 or Th22 cells in patients with low and high disease activity ((d) and (h), respectively), or with the presence or not of rheumatoid factor ((e) and (i), respectively), are also shown. Horizontal lines (in (d), (e), (h), and (i)) correspond to the median and *Q*_1_-*Q*_3_ interquartile range. RF: rheumatoid factor; ns: nonsignificant.

We then carried out an analysis of association between the levels (percentages and absolute numbers) and different clinical features or routine laboratory data of the patients with RA. We observed a significant association between the percent of pTh17 lymphocytes and Th22 cells in patients with RA (*r* = 0.63, *p* < 0.01, Figure 3(a)) but not in healthy controls (*r* = 0.28, *p* > 0.05, data not shown). However, no significant associations were detected between the number or proportion of pTh17 cells and different clinical parameters, including the time of disease duration, the severity of the disease (DAS28), the type of the disease (seropositive or not), or the doses of antirheumatic drugs prescribed (*p* > 0.05 in all cases, Figure 3 and data not shown). Likewise, no apparent associations were observed between the numbers or percentages of pTh17 cells and clinical laboratory parameters, including the titers of RF or ACCP antibodies, the levels of C reactive protein, or the erythrocyte sedimentation rate, among others (*p* > 0.05 in all cases, data not shown). In addition, similar negative results were observed in the case of the number or the proportion of Th22 lymphocytes, with no significant associations with the clinical or laboratory parameters tested (Figure 3 and data not shown).

### 3.3. In Vitro Induction of Pathogenic Th17 Cells

**Figure 4:**
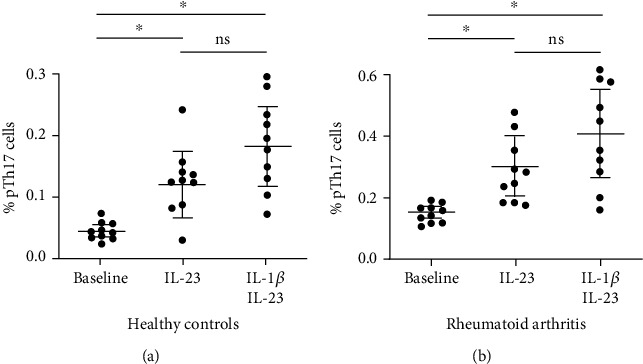
*In vitro* induction of pathogenic Th17 cells. PBMC from healthy controls and patients with rheumatoid arthritis were incubated in the presence or not of IL-23 or IL-1*β* plus IL-23 and then the percent of pathogenic Th17 (pTh17) cells, referred to total lymphocytes, and were analyzed by flow cytometry, as described in Material and Methods. Data correspond to the arithmetic mean and SD of ten separate experiments. ^∗^*p* < 0.05. ns: nonsignificant (Kruskal-Wallis test with Dunn's posttest).

We also assessed the possible effect of the cytokine IL-23, alone or in combination with IL-1*β*, on the *in vitro* induction of pTh17 lymphocytes. As shown in Figure 4, IL-23 induced a significant increase in the levels of pTh17 cells in both, patients and controls (*p* < 0.05, in both cases, compared to baseline levels). Similar results were observed when cells were stimulated with IL-23 plus IL-1*β* (Figure 4). Accordingly, the *in vitro* levels of induction (with IL-23 alone or with IL-23 plus IL-1*β*) of this subset of Th17 lymphocytes were similar in cell cultures from controls and patients with RA (Figure 4).

## 4. Discussion

Different reports have indicated that Th17 cells have a relevant role in the pathogenesis of RA and suggest that these lymphocytes and the cytokines released by them (mainly IL-17A) are a plausible therapeutic target in this and other immune-mediated inflammatory diseases [6, 17]. However, the flow cytometry analysis of the levels of Th17 cells in these studies has not been consistent and has usually been different from what was originally described [12, 23]. Therefore, in this study, we decided to reevaluate the levels of Th17 cells in patients with RA employing an extended immune phenotype and multiparametric flow cytometry analysis. Our data are in agreement with previous reports, showing increased levels (percentages and absolute numbers) of total Th17 (defined as CD4^+^IL-17^+^) and pTh17 cells (defined in this study as CD4^+^CD183^+^IL-17^+^IL-22^+^CD243^+^CD161^+^IFN-*γ*^+^IL-10^−^) in patients with RA [3, 6–9]. Nevertheless, we also observed similar increased levels of npTh17 cells in these patients, an unexpected finding since it has been proposed that this lymphocyte subset mainly exerts a homeostatic and regulatory effect [9, 15]. We consider that these data suggest that patients with RA have an increased induction of differentiation of Th0 lymphocytes into conventional (CD4^+^IL-17^+^) Th17 cells, which in turn results in enhanced numbers of both, pTh17 and npTh17 lymphocytes, with an apparent functional predominance of the pathogenic cells. Conversely, it is feasible that, in these patients, the Th17 cells with the nonpathogenic phenotype may also exert an unexpected proinflammatory effect, contributing thus to the pathogenesis of this condition. In this regard, it is of interest the report of Dankers et al., showing that the different subsets of memory Th17 cells (defined by their expression of the chemokine receptors CCR6, CXCR3 and CCR4, and isolated from the peripheral blood) exhibit a similar capability to induce the activation of synovial fibroblasts, making feasible the involvement of all them in the pathogenesis of the inflammatory phenomenon [25]. In addition, it is also of interest the significant negative association detected by Edavalath et al. between the levels of Th17 cells and disease activity in patients with RA [7].

In this study, we were unable to detect any significant association between the levels (either as absolute numbers or as percentages) of pTh17 cells and clinical or laboratory data. In this regard, it is feasible that due to the limited number of patients analyzed by us (*n* = 30), the correlation analyses did not have enough statistical power to detect significant associations, in no case. However, it is also possible that no significant associations exist between the levels of pTh17 cells, according to the extended phenotype analyzed in this study, and the different clinical or laboratory parameters of interest. Therefore, we consider that it would be important to carry out a prospective study with a large number of patients with RA to elucidate the exact relationship between the levels of pTh17 cells in peripheral blood and disease evolution.

We consider that the inclusion of markers such as MDR-1/CD243 into the phenotype of pTh17 cells is of interest since this membrane transporter is able to mediate the efflux of different chemical compounds (including some immunosuppressive and anti-inflammatory drugs), increasing the resistance of these cells to the antirheumatic therapy [23, 31, 32]. Moreover, the inclusion of the synthesis of IFN-*γ* and the lack of production of IL-10 as markers of pTh17 cells are another features of the phenotype analyzed by us, which is clearly related to the proinflammatory activity of these cells. Therefore, we consider that it would be of interest, as stated above, to reevaluate the possible association (causal?) between the levels of Th17 lymphocytes (CD4^+^IL-17^+^, pTh17, and npTh17) and different disease parameters in patients with RA, including those with early disease and naïve to antirheumatic drugs as well as in patients with established disease and under therapy.

Our data on the enhanced levels of Th22 lymphocytes in patients with RA are in agreement with previous reports on the detection of CD4^+^IL-22^+^IL-17^−^ cells in these patients [30, 33–35]. In this regard, Zhang et al. and Zhang et al. observed an increased proportion and absolute number of these lymphocytes in the peripheral blood from patients with RA as well as a significant association between the percentages of Th22 cells and disease activity [33, 34]. Likewise, it has been reported that patients with RA show an increased frequency of CD4^+^IL-22^+^IL-17^−^IFN-*γ*^−^ lymphocytes, which also correlated with disease activity [35]. Furthermore, it has also been informed that the therapy with disease-modifying drugs (leflunomide and methotrexate) diminished the increased levels of peripheral blood Th22 cells and serum IL-22 in patients with RA, which also correlated with diminution in disease activity [28]. Therefore, all these data suggest the involvement of Th22 cells in the pathogenesis of RA, a point that is supported by the potential proinflammatory effect of IL-22 as well as the pathogenic role of these lymphocytes in other chronic inflammatory conditions, including psoriasis, multiple sclerosis, or autoimmune thyroid disease [12, 27, 29, 36]. However, the lack of a significant association between the levels of Th22 (either the percentage or the absolute number) and clinical or laboratory parameters observed in our study remains as an interesting point to be analyzed in future studies.

According to our results on the raised levels of pTh17 cells in RA, we hypothesized that the lymphocytes from these patients should show an increased capability to differentiate into these pathogenic cells. Accordingly, we tested the *in vitro* differentiation of pTh17 cells, induced by IL-23 or by IL-1*β* plus IL-23. However, we observed similar levels of induction of pTh17 cells by these cytokines in samples from healthy controls and patients with RA. We consider that these results strongly suggest that there are additional factors that contribute to the *in vivo* induction of pTh17 lymphocytes in these patients. We also think that this information could be useful for the design of future immunosuppressive therapies employing biological blockers of proinflammatory cytokines in RA. In this regard, it would also have been of interest to analyze the *in vitro* induction of nonpathogenic Th17 lymphocytes, mainly by using TGF-*β*, an experimental condition not tested in this study.

## 5. Conclusions

Our data indicate that patients with RA show enhanced blood levels of the different subsets of Th17 cells (total Th17, pTh17, and npTh17) and Th22 lymphocytes tested in this study with no apparent association with clinical or laboratory parameters. Therefore, our data suggest that the levels of these cell subsets are not useful markers of disease evolution in patients with RA. However, it should be taken into account that one of the limitations of this study is the low number of individuals included in it.

## Figures and Tables

**Table 1:** Characteristics of patients with rheumatoid arthritis and healthy controls included in the study.

	RA patients	Controls
*n*	30	16
Age (years)	49.5 ± 9.5	47.5 ± 10.3
Sex (female/male)	28/2	15/1
Time evolution of disease (years)	7.8 ± 4.9	
Disease activity		
DAS28 ≤ 4	17	
DAS28 < 4	13	
Treatment		
MTX	11	
MTX + PDN	11	
MTX + PDN + SSZ	8	
Rheumatoid factor (+/-)	25/5	
ACCP (U/ml)	58 ± 28.4^∗^	
CH50 (U/ml)	30.6 ± 2.9^∗∗^	

DAS28: Disease Activity Score 28; MTX: methotrexate; PDN: prednisone; SSZ: sulfasalazine; RF: rheumatoid factor; ACCP: anticyclic citrullinated peptide antibodies (0.0-20.0); CH50: serum hemolytic complement levels. Data corresponds to the arithmetic mean ± SD. ^∗^Reference range: 0.0-20.0 U/ml. ^∗∗^Reference range: 16.1-31.2 U/ml.

**Table 2:** Summary of comparisons and *p* values of the levels of the different T cell subsets analyzed in blood samples from patients with RA and healthy controls.

Parameter compared (RA patients vs. controls)	Levels in RA	*p* value
Total Th17 cells (percentages)	Increased	<0.005
Total Th17 cells (absolute numbers)	Increased	<0.05
Pathogenic Th17 cells (percentages)	Increased	<0.005
Pathogenic Th17 cells (absolute numbers)	Increased	<0.005
Nonpathogenic Th17 cells (percentages)	Increased	<0.05
Nonpathogenic Th17 cells (absolute numbers)	Increased	<0.05
CXCR3^+^ Th22 cells (percentages)	Increased	<0.005
CXCR3^+^ Th22 cells (absolute numbers)	Increased	<0.005
CXCR3^−^ Th22 cells (percentages)	Increased	<0.05
CXCR3^−^ Th22 cells (absolute numbers)	Increased	<0.05

## Data Availability

The data underlying this article will be shared on reasonable request to the corresponding author.
